# Transiently Reduced PI3K/Akt Activity Drives the Development of Regulatory Function in Antigen-Stimulated Naïve T-Cells

**DOI:** 10.1371/journal.pone.0068378

**Published:** 2013-07-11

**Authors:** Eloho Etemire, Marco Krull, Mike Hasenberg, Peter Reichardt, Matthias Gunzer

**Affiliations:** 1 University Duisburg-Essen, University Hospital, Institute for Experimental Immunology and Imaging, Essen, Germany; 2 Otto von Guericke University, Institute of Molecular and Clinical Immunology, Magdeburg, Germany; Johannes Gutenberg University of Mainz, Germany

## Abstract

Regulatory T-cells (T_regs_) are central for immune homeostasis and divided in thymus-derived natural T_regs_ and peripherally induced iT_reg_. However, while phenotype and function of iT_regs_ are well known, a remarkable lack exists in knowledge about signaling mechanisms leading to their generation from naïve precursors in peripheral tissues. Using antigen specific naïve T-cells from mice, we investigated CD4+ CD25+ FoxP3- iT_reg_ induction during antigen-specific T-cell receptor (TCR) stimulation with weak antigen presenting cells (APC). We show that early signaling pathways such as ADAM-17-activation appeared similar in developing iT_reg_ and effector cells (T_eff_) and both initially shedded CD62-L. But iT_reg_ started reexpressing CD62-L after 24 h while T_eff_ permanently downmodulated it. Furthermore, between 24 and 72 hours iT_reg_ presented with significantly lower phosphorylation levels of Akt-S473 suggesting lower activity of the PI3K/Akt-axis. This was associated with a higher expression of the Akt hydrophobic motif-specific phosphatase PHLPP1 in iT_reg_. Importantly, the lack of costimulatory signals via CD28 from weak APC was central for the development of regulatory function in iT_reg_ but not for the reappearance of CD62-L. Thus, T-cells display a window of sensitivity after onset of TCR triggering within which the intensity of the PI3K/Akt signal controls entry into either effector or regulatory pathways.

## Introduction

Following T-cell receptor (TCR) triggering, naïve T-cells have multiple possibilities into which type of effector phenotype they develop [1]. Current concepts describe the effector lineages, Th1, Th2, Th17, T_FH_ and T_reg_ and variations of these, where the status of “lineage” is still debated [2]. For these T-cell types master regulators have been identified driving the expression of lineage-identifying functions [3]. Meanwhile evidence is accumulating that T-cells can express more than one “master regulator” and thereby acquiring new functions even after initial differentiation [4]. T_regs_ are a special lineage as they downregulate the activity of all other lines [5] and are divided into naturally occurring nT_reg_ generated from T-cell precursors in the thymus and induced iT_reg_, which form in the periphery by conversion of effector T-cells or by appropriate *de novo* activation of naïve T-cells [6]. T_regs_ can also be viewed based on their expression of the specific transcription factor FoxP3 as either FoxP3^+^ or FoxP3^−^ T_regs_ [7], [8].

Master regulators and functional capacities of established T-cell lineages are well understood [9] and very recently also the differences in signaling of established T_reg_ in response to TCR triggers have being elucidated in great detail [10]. However, much less is known about initial signaling events that lead to the generation of defined cell lineages. This is despite the fact that differentiation starts from a specific TCR trigger on naïve T-cells as common signal and only differs in “environmental conditions” such as the type of cytokines present or the APC present during triggering. Thus, next to TCR signaling the impact of environmental factors should trigger additional distinct events that can modulate the overall outcome of the effector function. In a way analogous to the identification of master regulators in stably established lineages [11] it should therefore be possible to identify the earliest signaling events differing in TCR-triggered T-cells on their way to specific lineages by investigating signaling pathways downstream of the TCR under distinct inducing conditions. Environmental conditions transforming naïve T-cells into specific lineages *in vitro* are well known. Next to TCR-triggering they require specific lineage inducing cytokines [11]. *In vitro* conditions for iT_reg_ induction typically involve TGFβ [12], [13] and iT_regs_ can be induced *in vivo* from naïve T-cells by targeting cognate antigens to immature dendritic cells (DC) [14], [15].

We previously demonstrated the induction of CD4+ CD25+ Foxp3- T_reg_ cells from TCR-transgenic T-cells *in vitro* without lineage-modifying cytokines using TCR-triggers by weak antigen presenting cells (i.e. non-professional APCs with low levels of costimulation such as naïve B-cells [16]). This approach allowed the transformation of naïve T-cells within 3 days of co-culture into FoxP3^−^ iT_regs_ that potently inhibited transplanted heart rejection *in vivo*. In contrast, when using mature DC as APC the cells developed into Th2-type effector cells (T_eff_) [16], [17]. In this model iT_regs_ demonstrated proliferation and expression of the activation markers CD25/CD69, yet in contrast to T_eff_ retained high surface levels of CD62-L [16]. Since these distinct phenotypes developed from a population of naïve T-cells within 72 hours of co-incubation, we reasoned that this model would allow studying the immediate signaling events leading to the generation of either T_eff_ or iT_reg_.

We report here that naïve T-cells on their way to iT_regs_ display remarkably similar signaling mechanisms compared to T-cells on their way to T_effs_. However, after an initial CD62-L loss similarly to T_eff_, iT_reg_ start re-expressing CD62-L 24 hours after onset of TCR-triggering. Low co-stimulatory levels from weak APC resulted in defective activation of the phosphatidyl-inositol-3-kinase (PI3K) pathway inducing a transiently lower activity of Akt accompanied with increased expression of the Akt-hydrophobic motif-specific phosphatase PHLPP1 in iT_regs_. The regulatory phenotype could be overridden by external CD28 stimulation. Importantly, later PI3K signaling in iT_reg_ reached again effectivity levels of T_eff_. Thus, transiently reduced PI3K activity in the first 24 hours of TCR triggering appears to be decisive in the lineage decision of iT_regs_ in this model.

## Results

### Transient Downregulation of CD62-L is Mediated by TACE

iT_regs_ generated by antigen specific activation of naïve T-cells through naïve B-cells (TofB) were identified by high expression of CD62-L despite proliferation and expression of the activation markers CD25 and CD69 [16]. This was in contrast to T_effs_ generated from activating naïve T-cells by DC (TofDC) that showed permanent downregulation of CD62-L and effector functions of conventionally activated T-cells [16]. This system provided a model for investigating the molecular mechanisms driving T-cell differentiation from naïve T-cells as common starting point.

**Figure 1:**
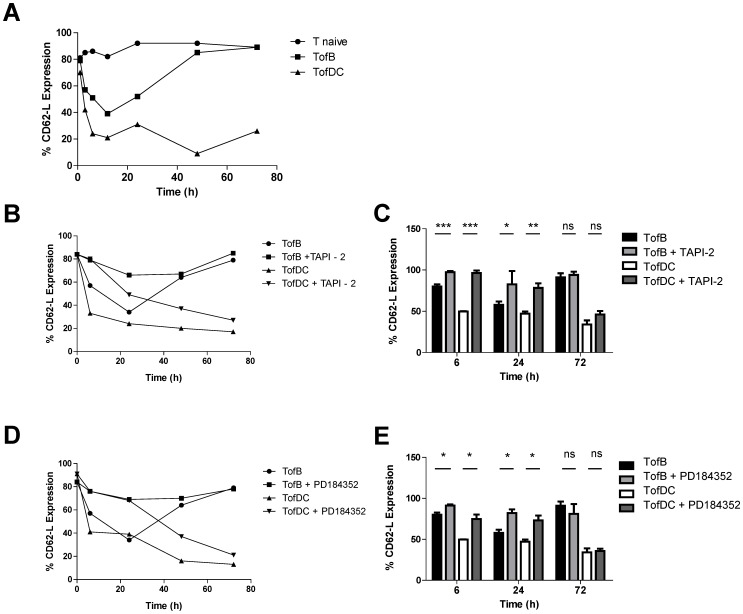
Downregulation of CD62-L is mediated by TACE. Naïve antigen specific T-cells were either left untreated (T naïve) or stimulated with either naïve B-cells (TofB) or activated dendritic cells (TofDC), both loaded with a cognate peptide of chicken ovalbumin, for different periods of time and in the absence or presence of inhibitors. Subsequently the number of cells expressing CD62-L was measured by flow cytometry. (A) one representative Kinetic of CD62-L expression on naïve and differentially activated T-cells over a 72 hour incubation time (representative experiment of 5) (B) Kinetics of CD62-L expression in the presence of the TACE sheddase inhibitor, TAPI-2. (C) The inhibition of TACE shows a significant interference with CD62-L shedding up to 24 hours after the initiation of T-cell priming. (D) Kinetics of CD62-L expression in the presence of the MEK-inhibitor PD184352 (E) Inhibition of MEK blocks CD62-L shedding in the first 24 hours of T-cell activation while the long term regulation of CD62-L expression is independent of MEK-TACE driven regulation. (C) and (E) show mean values+SEM of three independent experiments.

To clarify when loss of CD62-L on T-cells started relative to the onset of APC contact we observed the levels of surface CD62-L on T-cells following co-incubation with naïve B-cells or DC. Interestingly, under both conditions we observed initial CD62-L shedding that was more extensive in TofDC at 6 hours after contact formation but reached almost the same level in TofDC and TofB after 12 hours (Figure 1A). However starting at 24 hours after contact formation, TofB re-expressed CD62-L, and at 72 hours the number of CD62-L expressing cells was undistinguishable from naïve T-cells cultured alone. In contrast, CD62-L in TofDC was lost from most T-cells at 12–72 hours of observation (Figure 1A). It is pertinent to note that based on gating strategy and morphological analysis, we can exclude differential apoptosis kinetics as driving this difference in CD62-L kinetics (data not shown). Thus, CD62-L shows distinct kinetics in T-cells developing into iT_reg_ or T_eff_ cells.

CD62-L shedding marks T-cell activation and is mediated by the matrix metalloprotease TACE on the cell surface [18]. To clarify if TACE was involved in early CD62-L loss here, we blocked TACE with the specific inhibitor TAPI-2. Indeed TAPI-2 significantly reduced CD62-L shedding in TofB and TofDC at 6 and 24 hours of co-incubation but importantly not at 72 hours (Figure 1B–C).

TACE activity is linked to TCR triggering by the signaling effector MEK, which in turn activates the kinase Erk. Erk phosphorylates the inactive endoplasmatic TACE leading to its extracellular expression [19], [20]. To test, whether Erk was also involved in CD62-L shedding here we inhibited MEK using PD184352. Confirming an important role of TACE in the early but not late loss of CD62-L we observed that MEK-inhibition induced a significant block of CD62-L shedding during the “shedding phases” at 6 and 24 hours of T-APC co-incubation (Figure 1 D–E). Taken together, CD62-L shedding in the first 6–24 hours of T-cell activation is a TACE mediated pERK driven event requiring antigen specific activation but being independent of the APC strength.

### Sustained Downregulation of CD62-L Mediated by PI3K and mTOR

The observation that TACE drives only the initial regulation of CD62-L required to identify pathways responsible for the long-term regulation of CD62-L in TofB and TofDC. The PI3K/mTOR pathway is involved in regulating CD62-L following TCR triggering by sequestration of the transcription factor Foxo1 from the nucleus. This leads to decreased expression of the transcription factor KLF2 which binds the promoter of CD62-L [10], [21], [22]. Having seen the different long-term expression of CD62-L in TofB and TofDC we hypothesized that differences in PI3K/mTOR signaling might provide a mechanism for this dichotomy. We thus investigated the involvement of both pathways on protein and mRNA levels of CD62-L and KLF-2.

**Figure 2:**
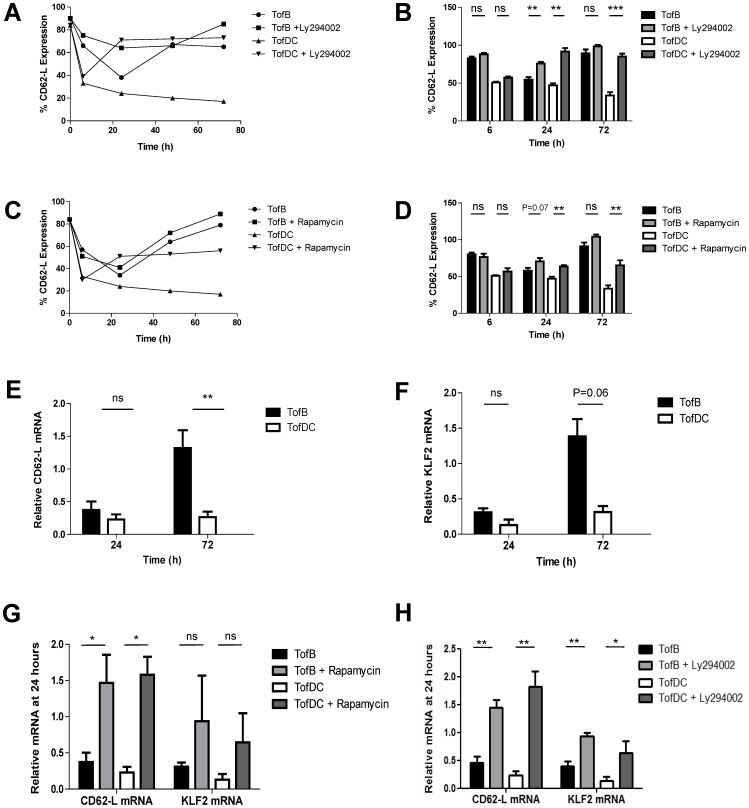
Sustained downregulation of CD62-L is mediated by PI3K and mTOR signaling. For FACS analysis, cells were treated and analyzed as described in Figure 1, while for qPCR analysis T-cells were recovered/enriched from co-cultures by MACS depletion. (A) Kinetics of CD62-L expression in the presence of the PI3K inhibitor, LY294002. (B) LY294002 has no effect on CD62-L loss in the first 6 hours after onset of T-cell activation but induces significant upregulation of CD62-L at later time points. (C) Kinetics of CD62-L expression in the presence of the mTOR inhibitor, Rapamycin. (D) Rapamycin has no effect on CD62-L loss in the first 6 hours after onset of T-cell activation but induces upregulation of CD62-L at later time points. (E) qPCR analysis of CD62-L mRNA expression level in TofB and TofDC at 24 and 72 hours. (F) qPCR analysis of KLF2 mRNA expression levels in TofB and TofDC at 24 and 72 hours. (G) qPCR analysis of CD62-L mRNA induction in both TofB and TofDC by Rapamycin and a detectable yet non-significant induction of KLF2 mRNA. (H) qPCR analysis of CD62-L and KLF2 mRNA induction in TofB and TofDC in the presence or absence of Ly294002. Data in B and D-H are means+SEM of 3–5 independent experiments.

The addition of the PI3K inhibitor Ly294002 (10 µM) to both co-culture systems had no effect on early CD62-L regulation. However, at 24 hours CD62-L surface expression was significantly increased in TofB and TofDC. Importantly, at 72 hours, PI3K inhibition significantly increased CD62-L in TofDC but not in TofB (Figure 2 A–B). The addition of the mTOR inhibitor Rapamycin (100 nM) partially mirrored the effects of PI3K inhibition in TofDC, but not in TofB. We saw no significant effect of Rapamycin on CD62-L surface expression at any time in TofB while in TofDC the inhibitor significantly increased the expression at 24 and 72 hours, albeit to a lower extent as the PI3K-inhibitor (Figure 2 C–D). The effects of Rapamycin and LY2924002 were titratable but both inhibitors showed toxic effects when used at supra-effective doses during long-term exposure (Figure S1A and Figure S1B).

Re-expression of CD62-L is dependent on protein neo synthesis [22]. Consequently we measured significantly higher levels of mRNA for both, CD62-L (Figure 2E) and KLF-2 (Figure 2F) in TofB as opposed to TofDC by qPCR. Already detectable as a trend at 24 hours, it was more pronounced at 72 hours. The inhibition of PI3K (Figure 2G) or mTOR (Figure 2H) also led to a pronounced increase of mRNA for CD62-L and KLF-2 in both TofB and TofDC at 24 hours after initiation of activation.

Collectively this suggests that the induction of TofB and TofDC is similar at the level of initial CD62-L shedding but shows differences with respect to PI3K/mTOR signaling culminating in high versus low levels of CD62-L at later time points.

### T-cells Triggered by iDC Replicate CD62-L Expression and Regulatory Behavior of TofB

While naïve B-cells are able to activate naïve T-cells in culture, it is unlikely that this happens in lymph nodes *in vivo*. Here, B-cells are localized in follicles and the majority of naïve T-cells localize in T-cell zones enriched in DC [23]. While DC are the most efficient APC under inflammatory conditions, their lack leads to autoimmunity rather than immune suppression [24]. Thus, without inflammation DC serve as gate keepers of self tolerance [14], [25]. Here DC reside in a resting or immature state in the middle of T-cell zones maintaining self tolerance by the generation of iT_regs_ [26] *in vivo*. Thus, immature DC (iDC) should be a more physiological weak APC to investigate the plasticity of T-cell responses in our model.

Therefore we generated iDC by culturing non-adherent bone marrow cells in the presence of GM-CSF alone as described [27]. iDC in comparison to DC expressed comparable levels of MHC II but far lower levels of CD11c and the co-stimulatory molecules CD80 and CD86 (Figure S2).

**Figure 3:**
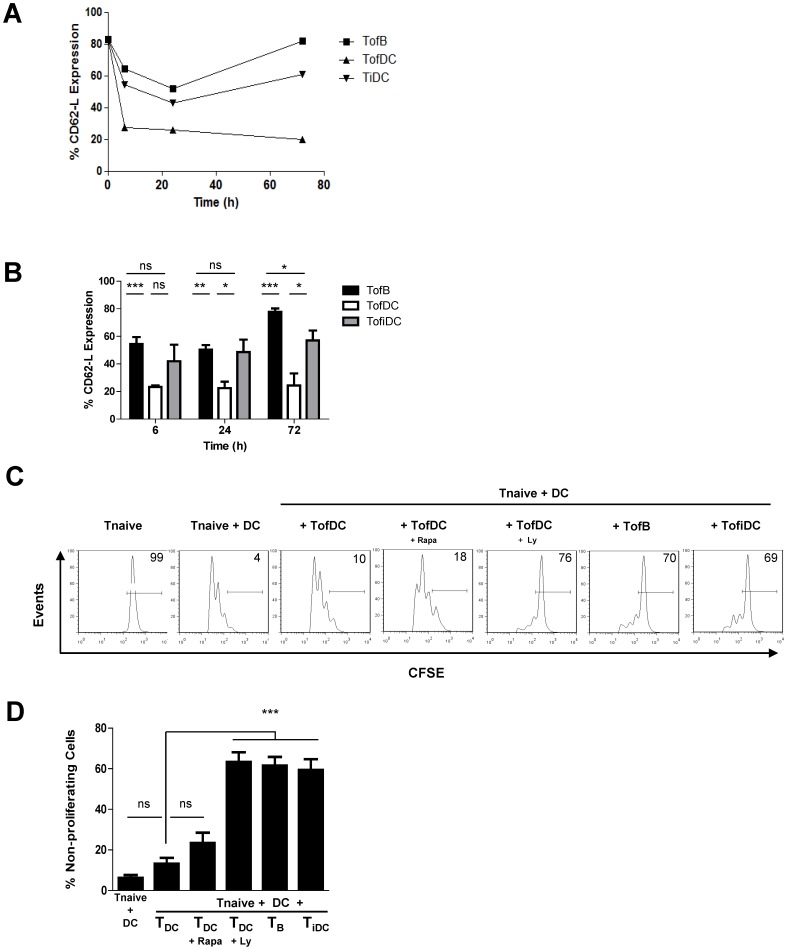
T-cells triggered by immature DC replicate CD62-L expression signature and regulatory behavior of TofB. Naïve antigen specific T-cells were treated as described in Figure 1 except for priming with immature DC (TofiDC), which was additionally run in a separate culture. For *in vitro* inhibitory assays, naïve T-cells were stained with CFSE and incubated alone or in the presence of mature dendritic cells loaded with cognate peptide. To the same type of culture we also added various types of CFSE-negative activated T-cells for modulation of T-cell proliferation. The proliferation of the naïve T-cells was measured by CFSE-dilution after 72 hours of co-incubation (A) Kinetics of CD62-L dynamics in TofiDC with respect to the TofB and TofDC (B) CD62-L levels in naive T-cells primed with iDC, B-cells or mature DC. (C) Regulatory capacity of the various activated T-cells (representative experiment). (D) Statistical analysis shows that TofB, TofDC_Ly_ and TofiDC induce significant inhibition of naive T-cell proliferation relative to TofDC while TofDC_Rapa_ induce no significant inhibition. Data are means+SEM of 4–5 experiments.

Next we measured CD62-L expression in T-cells primed by iDC (TofiDC). Remarkably we found a transient downregulation and re-expression of CD62-L in TofiDC in a fashion almost identical to that seen with TofB. Furthermore, TofiDC always showed a higher expression of CD62-L as compared to TofDC (Figure 3A–B). Thus it was important to test whether TofiDC would also functionally equal TofB. We therefore decided to test, whether TofiDC were functionally T_regs_. *In vitro* inhibitory tests indeed confirmed that TofiDC possessed regulatory activity of the same potency as TofB (Figure 3C–D). In addition, the characterization of TofiDC showed them to be Foxp3 negative just as TofB (data not shown).

The analysis of the PI3K/mTOR pathway had suggested that an incomplete triggering of this mechanism mediated the inability to permanently downregulate CD62-L in TofB and TofDC. We therefore asked, whether the blockade of this pathway would also functionally change the activity of TofDC. Thus we generated TofDC in the presence of Ly294002 and Rapamycin. When tested in *in vitro* inhibitory assays, indeed PI3K inhibited cells (TofDC+Ly) presented with a regulatory capacity equal to TofiDC and TofB (Figure 3 C–D). Interestingly, the blockade of mTOR could not significantly induce a regulatory phenotype in TofDC (Figure 3C–D) despite its pronounced effect on CD62-L expression. Thus, incomplete PI3K- but not mTOR-activation was responsible for the induction of iT_regs_ in this system.

### Sub-optimal Co-stimulation Induces Regulatory Function but does not Influence CD62-L

Naïve B-cells and iDC both lack costimulatory molecules, especially CD80 and CD86 (Figure S2 and [16]). These molecules are triggers for CD28 on T-cells which in turn is a prominent activator of the PI3K pathway [28]. The lack of costimulation might thus underly the inability of naïve B-cells or iDC to induce effector T-cells.

**Figure 4:**
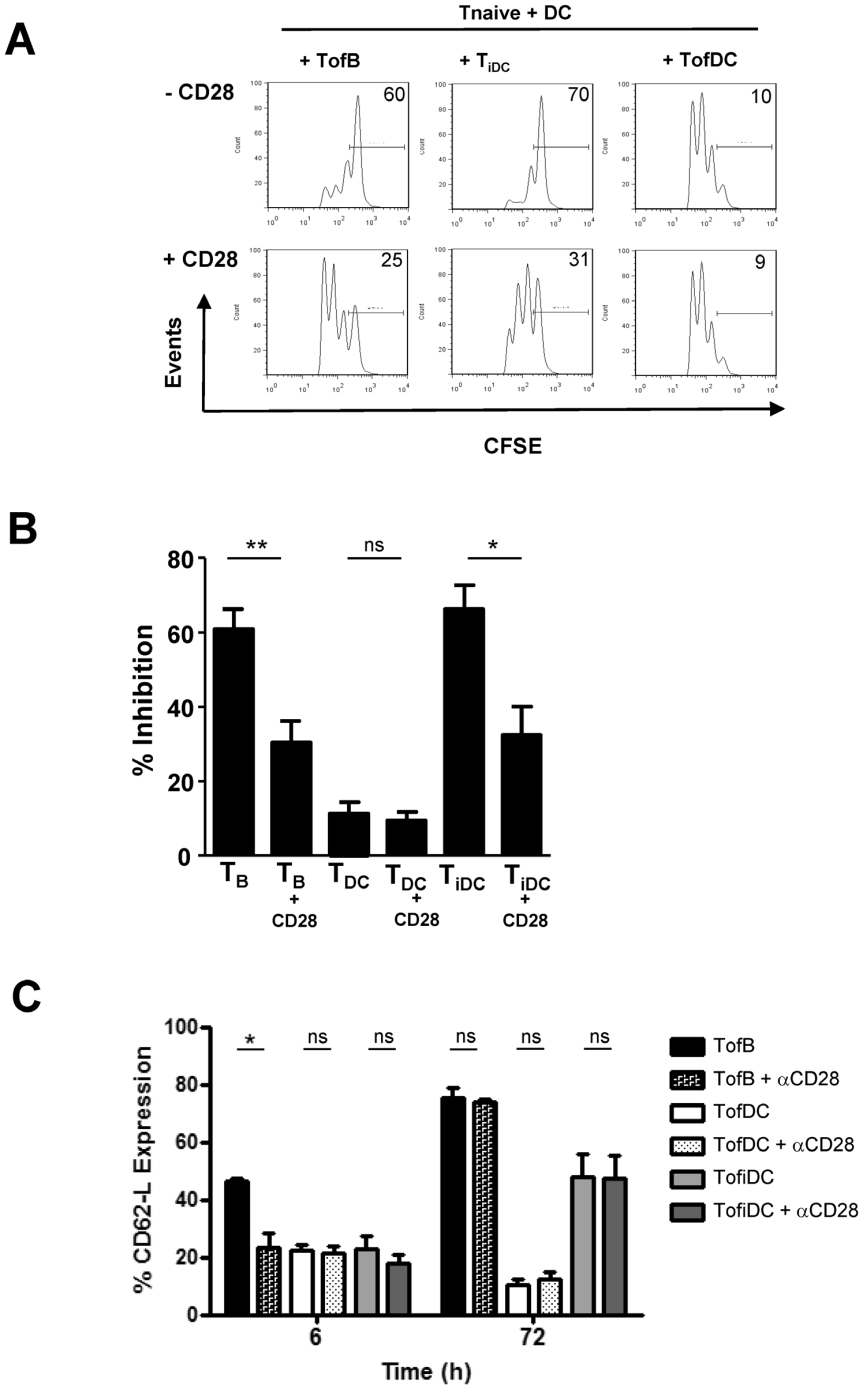
Sub-optimal co-stimulation drives acquisition of regulatory function but exerts negligible influence on CD62-L dynamics. Co-stimulation was augmented by the addition of CD28 antibodies to cultures during the generation of TofB, TofiDC or TofDC for their use in downstream studies. The obtained activated cells were then tested for their inhibitory capacity against naïve T-cells. (A) Representative FACS analysis of *in vitro* proliferation assays of CFSE stained naïve T-cells in the presence of various activated T-cells showing that the addition of CD28 antibodies during the generation of TofB and TofiDC strongly reduces their regulatory capacity. (B) Statistical analysis of the effect of increased co-stimulation on the regulatory capacity of TofB and TofiDC. (C) CD62-L expression levels in T-cells generated with or without additional CD28 antibodies. Data are means+SEM of 3–4 experiments.

To study this we generated TofB, TofDC and TofiDC in the absence or presence of artificial CD28-signals provided by soluble antibodies. In an *in vitro* test for T-cell activation we observed that TofB and TofiDC generated in the presence of CD28 antibodies showed significantly less inhibition of T-cell proliferation as compared to TofB or TofiDC generated without additional co-stimulation (Figure 4 A–B). TofDC generated with additional CD28 triggering had the same effect on T-cell proliferation as the regular TofDC indicating the level of stimulation in DC could not be further augmented by the addition of CD28. However, opposed to the fundamental change in functional activity induced by CD28 co-ligation we only observed a mild effect of this treatment on CD62-L loss at 6 hours in TofB while at later time points CD62-L expression returned to its high levels seen before with naïve B-cells or iDC (Figure 4 C).

Together these results suggest that defective CD28 co-stimulation plays a key role in the induction of regulatory function in naïve T-cells but has no discernible influence on CD62-L dynamics.

### Defective Akt Signaling Following T-cell Triggering by Weak APCs

Analysis so far had suggested suboptimal PI3K/mTOR signaling as basis for the induction of iT_regs_ by weak APC. However, TofB and TofiDC were viable proliferating cells expressing levels of CD25 equivalent to TofDC (not shown). Proliferation of T-cells requires nutrient production. This is dependent on a functional PI3K/mTOR pathway and mediated by the downstream effector Akt [22]. Thus the question was how a low activity of the PI3K/mTOR pathway would be consistent with active cell proliferation.

**Figure 5:**
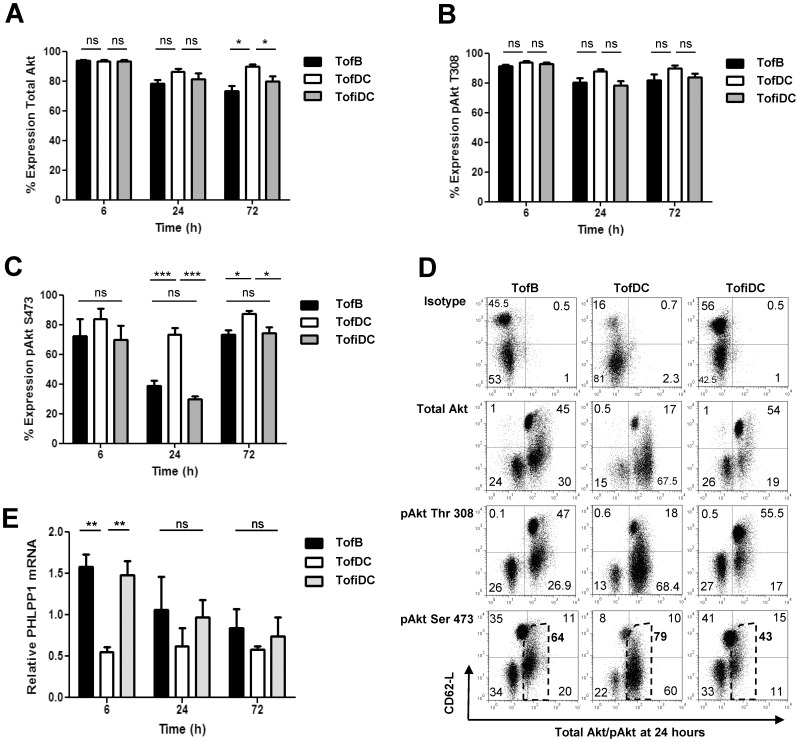
Phospho-site specific defective Akt signaling observed following T-cell triggering by weak APCs. Cells were treated as described in Figure 3 including priming with immature DC (TofiDC) and intracellular FACS staining of total Akt and phopho-forms was performed at indicated time points. For qPCR analysis, T cells were recovered/enriched from co-cultures by MACS depletion. (A) Time course of the number of cells expressing total Akt. (B) Time course of the number of cells expressing pAkt Thr308. (C) Time course of the number of cells expressing pAkt Ser473. (D) One representative FACS blot showing reduced pAkt Ser473 activation in TofB and TofiDC at 24 hours. Numbers in quadrants denominate the percentage of cells. The dashed region indicates the pSer473 positive fraction analyzed for its MFI value (fat number on the right of the dashed region). (E) qPCR readout of PHLPP1 levels in TofB and TofiDC compared to TofDC. Data are means+SEM of 3–4 experiments. Data in D are representative of 4 independent experiments.

Therefore we tested the activity of Akt via the phosphorylation status of its activation loop at Thr308 and the hydrophobic motif at Ser473. Differences at the phosphorylation of Ser473 can be found in T_regs_ [10], [29]. As expected, in all three T-cell types total levels of Akt were mostly unchanged over the observation period of 72 hours (Figure 5A and Figure S3). However, also Thr308-phosphorylation was always identical in all cells (Figure 5B and Figure S3). But we did detect defective phosphorylation of Ser473 in TofB and TofiDC compared to TofDC starting at 6 hours after contact initiation and being highly significant at 24 hours (Figure 5C/D). Both, the number pAkt Ser473-expressing cells as well as their amount of pAkt Ser473 (by MFI) were lower in TofB/TofiDC as compared to TofDC (Figure 5D). However, at 72 hours the reduced phosphorylation of Ser473 was mostly lost again (Figure 5C and Figure S3B). Thus during generation, TofB and TofiDC have a phase of reduced Akt-Ser473 phosphorylation that is gradually abrogated again.

What mechanism could drive this phospho-site specific defect in TofB and TofiDC? We measured the phosphatase PHLPP1 which can induce regulatory properties in T-cells and is associated with the defective phosphorylation of pAkt Ser473 [12]. Indeed, PHLPP1 mRNA was significantly increased in TofB and TofiDC as compared to TofDC. This was highly significant at 6 hours and still maintained as a trend at 24 and 72 hours (Figure 5E). These levels of PHLPP1 fit with the temporal downregulation of pAkt Ser473. Collectively these data show that during the generation of iT_regs_ by weak APC there was an initial phase of weak PI3K/mTOR induction leading to transiently defective Akt activity and the re-expression of CD62-L coincided with this period of weak Akt activity.

## Discussion

By virtue of their unique activity as suppressors of all other T-cell types T_regs_ occupy a special niche in adaptive immunity. There are several studies exploring their biology ranging from their development/induction to the mechanisms of action [30], [31]. However, despite the extensive knowledge we have about these issues of T_reg_ biology there is a remarkable dearth of information regarding early signaling events driving the induction of iT_regs_ from naïve CD4 T-cells despite the central role of this mechanism in adaptive immunity.

To bridge this gap, we present in this report a kinetic study examining early signaling events that lead to the production of CD4+ CD25+ Foxp3- iT_regs_ from naïve CD4 T-cells. Thereby we took advantage of our previously reported model of naïve CD4 T-cell co-culture with naïve B-cells as APC [16]. In addition, we incorporated a well-known second type of weak APC in our system, immature DC, which also provides a physiologically relevant means of T-cell activation *in vivo*. Furthermore we performed experiments on 2 different strains of mice, with both showing similar results and trends thus negating the possibility of our observations been a strain specific effect.

We were surprised to find that the induction of T_regs_ in this model was achieved via triggering of remarkably similar pathways as that of T_effs_. We only found a transiently reduced activity of the PI3K/Akt pathway between 6–24 hours post-activation as a characteristic sign for the induction of a T_reg_ phenotype. This was accomplished by the transient induction of PHLPP1, an Akt hydrophobic motif-specific phosphatase, selectively in T_regs_. It is very interesting to see that a recent report found very similar defects in signaling (namely low Akt473 phosphorylation) in fully established FoxP3-expressing Tregs [10]. Thus, the signaling defect seen in T_regs_ might also be the same initial defect that leads to T_reg_ induction from naïve T-cells.

The role of the PI3K pathway in T_reg_ development is presently controversial as there are reports that PI3K activation inhibits T_reg_ development [32] while others claim the opposite [33]. Our data indicate that PI3K activation is necessary for and compatible with iT_reg_ development but only when being attenuated at the beginning of the T-cell stimulation. In our model, this is achieved by weak APCs such as naïve B-cells and immature DC via their characteristically low co-stimulatory input derived from sub-optimal surface levels of co-stimulatory molecules. Interestingly, while not a downstream effector molecule of the TCR complex, CD28 co-stimulation is known to be vitally involved in producing robust PI3K activation [34], [35]. Adding CD28 to the weak APC-T-cell co-culture led to the restoration of the effector phenotype and abrogation of regulatory function. This shows that a low degree of CD28 signaling at the onset of TCR triggering can lead to a signal sufficiently high to drive T-cell activation and differentiation but favoring the development of a regulatory phenotype, a conclusion also reached by others [36]. As PI3K activity reached levels similar to those in T_effs_ at timepoints later than 24 hours, early signaling events up to 24 hours after the initial TCR trigger appear to occur in a window of sensitivity in a T-cell, where the activity of the PI3K pathway is decisive in determining the later effector phenotype.

A characteristic peculiarity of iT_reg_ in our model was the high expression of CD62-L despite effective activation and proliferation of the cells. We found that CD62-L in iT_reg_ followed a pattern of initial TACE-mediated loss, as in T_eff_. Then, however, the cellular development deviated and iT_reg_ re-expressed CD62-L while T_eff_ maintained low levels of the molecule. Again we observed that CD62-L re-expression coincided with the timing of defective PI3K/Akt activity. Attenuation of PI3K signaling during T_reg_ differentiation/development has been put forward by Okkenhaug and coworkers as allowing for the nuclear return and subsequent binding of the transcription factor Foxo on the promoter region of the Foxp3 locus in the nucleus [33]. We extend this position by suggesting that Foxo on return possibly also binds onto the promoter for KLF2 thereby leading to increased CD62-L transcription and its re-expression in the iT_reg_ but not the T_eff_ in our model. The latter showed consistently high PI3K activity. It was possible that CD62L was additionally modulated by the selective presence of microRNAs as recently reported [37]. However, we could not confirm a role for the miRNA let7-b in inducing CD62-L re-expression as we found it to be equally downregulated in both TofB and TofDC (unpublished data). Also of interest was the observation that both T_regs_ in our model were Foxp3^−^. While we previously showed that the TofB developed independent of IL-10 presence in B cells, the Roncarolo group have shown that immature DCs produce Foxp3^−^ CD25^low^ Tr1 like T_regs_ under allogeneic sub-optimal T cell activation conditions under the influence of IL-10 [38].

Having observed the re-expression of CD62-L on the iT_regs_ in our model we asked whether the re-expression of CD62-L was always associated with the acquisition of regulatory function. This question is pertinent as there are several studies linking the CD62-L transcription factor Foxo with T_reg_ induction [39]–[42] or function [10]. However, CD62-L aside its requirement for homing to lymph nodes has also been related with the acquisition and maintenance of memory in central memory T-cells [43], [44] as well as with the development of effector function such as lytic activity in human tumor-infiltrating T lymphocytes [45]. Our data indeed showed independence of the development of lymphatic homing capacity and regulatory activity since Ly294002-treated T_eff_ with high CD62-L did show regulatory properties when tested functionally while Rapamycin-treated TofDC with high CD62-L showed no inhibitory function. Furthermore, our data also show that increased co-stimulation via CD28 has no discernible effect on CD62-L regulation while at same time inducing a complete loss of regulatory function.

Together these data suggest a compartmentalization in the signaling pathways controlling the acquisition of regulatory activity and homing. Obviously, only PI3K but not mTOR inhibition is able to upregulate CD62-L together with the acquisition of a regulatory phenotype in naïve T-cells. This unexpected finding is interesting given that administration of the mTOR-inhibiting drug Rapamycin is an established means of inducing T_reg_ expansion from CD4^+^ CD25^+^ cells [46]. Furthermore, Rapamycin was reported to induce T_regs_ from naïve T-cells [47]. In said study however, naïve CD4^+^ T-cells were co-cultured with B-cells as APC in the presence of Rapamycin. Based on similarities with our study, it is tempting to speculate that the resultant regulatory behavior might have been conferred by the presence of the B-cell, as in our system. However, even in the absence of B-cells, naïve T-cells triggered with CD3 antibodies in the presence of Rapamycin or Ly294002 do acquire a T_reg_ phenotype [48]. This disparity in results might be indicative that antigen specific activation by weak APC can trigger more delicate pathways of T-cell activation as opposed to the very strong stimulus coming from polyclonal CD3 crosslinking. The nature of these triggers has, however, remains elusive so far.

In summary, we present data showing that remarkably similar signaling pathways drive the generation of iT_regs_ and T_eff_ from naïve T-cells and iT_reg_ inducing signaling features are again very similar to those active in established T_reg_ [10]. The key difference between the induction of a T_reg_ and a T_eff_ phenotype appears to be signaling thresholds combined with carefully timed signal intensity modulation in the PI3K/Akt pathway_._ At the same time separate mechanisms exist, that control the expression of specific homing markers. A better understanding of these mechanisms might help to provide means for a selective generation and tissue specific recruitment of T-cell effector phenotypes in the future.

## Materials and Methods

### Ethics

All cells used in this study were isolated from organs of mice. Before organ harvest the mice were sacrificed painlessly by deep narcosis followed by cervical dislocation according to institutional guidelines and no invasive procedures were carried out using live animals. According to the German Tierschutzgesetz (TSchG) the use of animal tissue following painless sacrifice and without any further treatment of live animals is not considered an animal experiment and therefore does not require ethical approval. However, the animal welfare officer of both institutions, where the experiments were performed, was informed and had to collect information on the number of animals used for tissue donation. This information was forwarded to the local authorities (Landesverwaltungsamt Sachsen-Anhalt and LANUV, Nordrhein Westfalen).

### Mice

DO11.10 [49] and OT-II mice [50] with transgenic TCRs recognising a peptide of chicken-ovalbumin (pOVA AA323–339) were used for T-Cells while B-cells and bone marrow DCs (DCs) were obtained from C57BL/6 and BALB/c mice. Animals were housed and bred in an animal facility of the Otto-von Guericke University and the University Duisburg-Essen, Germany, under SPF-conditions and treated according to institutional guidelines.

### Cell Preparation

Naive CD4^+^ T-cells from spleens of DO11.10 or OT-II mice, splenic B-cells BALB/c or C57BL/6 and mature bone marrow derived DC were all generated as previously described [16] while immature DC (iDC) were generated using the same technique as DC but with absence of IL-4 as described [51]. All cells were cultured at 37°C with 5% CO_2_.

### T-cell Activation Assays

Naïve T-cells were co-cultured with OVA-peptide loaded LPS stimulated DC, iDC or naive B-cells at a ratio of 10∶1/10∶1 or 1∶1, respectively. The cell culture medium was RPMI-based and supplemented with 10% fetal calf serum (Gibco, Los Angeles, USA). After 72 hours co-cultures underwent immunomagnetic depletion of non-CD4 cells using the MACS system as described [16]. B-cell (TofB), iDC (TofiDC) or DC (TofDC)–primed T-cells (with or without pharmacological modulators) were extensively washed with PBS after immunomagnetic recovery to deplete any inhibitors and assayed to test their inhibitory capacity on naïve T-cells primed with pOVA loaded DCs (10∶1) at a ratio of 1∶1 (T primed: T naïve). To measure T-cell proliferation, naïve DO11.10 or OT-II T-cells were stained with 5,6-carboxyfluorescein-diacetate-succinimidyl-ester (CFSE, 0.5 µM; Molecular Probes, Leiden, Netherlands). Readouts for proliferation or activation markers were taken at indicated timepoints. T-cell activation was modulated pharmacologically using TAPI-2 (Calbiochem, Darmstadt, Germany, 100 µM), Ly294002 (Promega, Mannheim, Germany, 10 µM), PD184352 (Selleck Chemicals, Munich, Germany, 2 µM) [22] and Rapamycin (Calbiochem, 100 nM) [52]. Anti-CD28 (Beckton Dickinson, Heidelberg, Germany) mediated co-stimulation was provided at 10 µg/ml.

### Flow Cytometry

For surface staining, all antibodies were purchased from BD except for CD11c (Caltag, Burlingame, USA), MHCII (eBioscience, Frankfurt, Germany) and CD80/86 (Abcam, Cambridge, UK). For intracellular staining, FITC conjugated pan Akt, pAkt Thr308 and pAkt Ser473 with isotype controls were from Cell Signalling Technology (Frankfurt, Germany) and used with the BD cytoperm/cytofix with GolgiPlug kit. Events were acquired on a BD FACSCalibur™ and LSRFortessa™.

### Real-Time PCR

T-cells co-incubated with B-cells, iDC or DC were recovered via the MACS system to an average purity of 88%. Total RNA was isolated and cDNA synthesized using the RNeasy kit and the Quantitect Reverse-Transcriptase-kit (Qiagen, Cologne, Germany). Primers were reagent kits from Qiagen. Real-time PCR was performed on either the Qiagen RotorGene or ABI prism 7000, using the QuantiTect SYBR Green PCR Kit (Qiagen) or the Maxima SYBR Green/ROX qPCR Master Mix (2×) kit (Fermentas, St. Leon-Rot, Germany) in duplicates. C*_t_* values were averaged and normalized against C*_t_* values of β-Actin. Results were derived using the comparative Ct method (ΔΔC*_t_*).

### Statistical Analysis

Statistical significance was evaluated with student’s t-test using GraphPad Prizm 5 (GraphPad Software, San Diego, CA, USA). P-values <0.05 were considered significant. Data were expressed as means +/− SEM with 3 or more independent experiments performed.

## Supporting Information

Figure S1 **Effect of inhibitor Titration on CD62-L levels.** Naïve antigen specific T-cells were stimulated with either naïve B-cells (TofB) or activated dendritic cells (TofDC), both loaded with a cognate peptide of chicken ovalbumin, for different periods of time and in the absence or presence of inhibitors. Subsequently, CD62-L expression levels on T cells were measured by flow cytometry. (A) One representative FACS blot showing the dose dependent regulation of CD62-L in T cells by the PI3K inhibitor LY294002. (B) One representative FACS blot showing the dose dependent regulation of CD62-L in T cells by the mTOR inhibitor Rapamycin. Data are representative of 3 independent experiments.
(PDF)

Figure S2 **Characterization of Immature DCs against mature DCs.** Immature DC were characterized with respect to expression levels of relevant lineage and stimulatory marker molecules.(A) Representative FACS histogram showing expression levels of the indicated surface molecules. Dashed lines represent isotypes and solid lines indicate expression level of quantified molecules (B) Comparison of % surface expression of MHC II, CD11c, CD80 and CD86 between both cell phenotypes. (C) On a per cell basis, DC express more CD11c than immature DC while both cells possess equal amounts of MHC II. (D) On a per cell basis, DC express more co-stimulatory molecules than immature DC. Data are means+SEM of 2 experiments.
(TIF)

Figure S3 **Akt signaling profile at 6 and 72 hours.** Cells were treated as described in Figure 5. (A) Representative FACS blots showing Akt/pAkt levels in TofB, TofiDC and TofDC at 6 h (B) Representative FACS blots showing Akt/pAkt levels in TofB, TofiDC and TofDC at 72 hours.
(TIF)

## References

[1] Kanno Y, Vahedi G, Hirahara K, Singleton K, O’Shea JJ (2012) Transcriptional and epigenetic control of T helper cell specification: molecular mechanisms underlying commitment and plasticity. Annu Rev Immunol 30: 707–731. 22224760 10.1146/annurev-immunol-020711-075058 PMC3314163

[2] Zhu J, Yamane H, Paul WE (2010) Differentiation of effector CD4 T cell populations. Annu Rev Immunol 28: 445–489. 20192806 10.1146/annurev-immunol-030409-101212 PMC3502616

[3] Weaver CT, Hatton RD, Mangan PR, Harrington LE (2007) IL-17 family cytokines and the expanding diversity of effector T cell lineages. Annu Rev Immunol 25: 821–852. 17201677 10.1146/annurev.immunol.25.022106.141557

[4] Oestreich KJ, Weinmann AS (2012) Master regulators or lineage-specifying? Changing views on CD4(+) T cell transcription factors. Nat Rev Immunol 12: 799–804. 23059426 10.1038/nri3321 PMC3584691

[5] Sakaguchi S, Miyara M, Costantino CM, Hafler DA (2010) FOXP3+ regulatory T cells in the human immune system. Nat Rev Immunol 10: 490–500. 20559327 10.1038/nri2785

[6] Wood KJ, Bushell A, Hester J (2012) Regulatory immune cells in transplantation. Nat Rev Immunol 12: 417–430. 22627860 10.1038/nri3227

[7] Passerini L, Di NS, Gregori S, Gambineri E, Cecconi M, et al (2011) Functional type 1 regulatory T cells develop regardless of FOXP3 mutations in patients with IPEX syndrome. Eur J Immunol 41: 1120–1131. 21400500 10.1002/eji.201040909 PMC3107421

[8] Vieira PL, Christensen JR, Minaee S, O’Neill EJ, Barrat FJ, et al (2004) IL-10-secreting regulatory T cells do not express Foxp3 but have comparable regulatory function to naturally occurring CD4+CD25+ regulatory T cells. J Immunol 172: 5986–5993. 15128781 10.4049/jimmunol.172.10.5986

[9] O’Shea JJ, Paul WE (2010) Mechanisms underlying lineage commitment and plasticity of helper CD4+ T cells. Science 327: 1098–1102. 20185720 10.1126/science.1178334 PMC2997673

[10] Ouyang W, Liao W, Luo CT, Yin N, Huse M, et al (2012) Novel Foxo1-dependent transcriptional programs control T(reg) cell function. Nature 491: 554–559. 23135404 10.1038/nature11581 PMC3771531

[11] Szabo SJ, Kim ST, Costa GL, Zhang X, Fathman CG, et al (2000) A novel transcription factor, T-bet, directs Th1 lineage commitment. Cell 100: 655–669. 10761931 10.1016/s0092-8674(00)80702-3

[12] Patterson SJ, Han JM, Garcia R, Assi K, Gao T, et al (2011) Cutting edge: PHLPP regulates the development, function, and molecular signaling pathways of regulatory T cells. J Immunol 186: 5533–5537. 21498666 10.4049/jimmunol.1002126 PMC4015973

[13] Semple K, Nguyen A, Yu Y, Wang H, Anasetti C, et al (2011) Strong CD28 costimulation suppresses induction of regulatory T cells from naive precursors through Lck signaling. Blood 117: 3096–3103. 21245484 10.1182/blood-2010-08-301275 PMC3062312

[14] Kretschmer K, Apostolou I, Hawiger D, Khazaie K, Nussenzweig MC, et al (2005) Inducing and expanding regulatory T cell populations by foreign antigen. Nat Immunol 6: 1219–1227. 16244650 10.1038/ni1265

[15] Apostolou I, von Boehmer H (2004) In vivo instruction of suppressor commitment in naive T cells. J Exp Med 199: 1401–1408. 15148338 10.1084/jem.20040249 PMC2211808

[16] Reichardt P, Dornbach B, Song R, Beissert S, Gueler F, et al (2007) Naive B cells generate regulatory T cells in the presence of a mature immunological synapse. Blood 110: 1519–1529. 17392507 10.1182/blood-2006-10-053793

[17] Reichardt P, Dornbach B, Gunzer M (2007) The molecular makeup and function of regulatory and effector synapses. Immunol Rev 218: 165–177. 17624952 10.1111/j.1600-065X.2007.00526.x

[18] Moreau HD, Lemaitre F, Terriac E, Azar G, Piel M, et al (2012) Dynamic In Situ Cytometry Uncovers T Cell Receptor Signaling during Immunological Synapses and Kinapses In Vivo. Immunity 37: 351–363. 22683126 10.1016/j.immuni.2012.05.014

[19] Diaz-Rodriguez E, Montero JC, Esparis-Ogando A, Yuste L, Pandiella A (2002) Extracellular signal-regulated kinase phosphorylates tumor necrosis factor alpha-converting enzyme at threonine 735: a potential role in regulated shedding. Mol Biol Cell 13: 2031–2044. 12058067 10.1091/mbc.01-11-0561 PMC117622

[20] Soond SM, Everson B, Riches DW, Murphy G (2005) ERK-mediated phosphorylation of Thr735 in TNFalpha-converting enzyme and its potential role in TACE protein trafficking. J Cell Sci 118: 2371–2380. 15923650 10.1242/jcs.02357

[21] Fabre S, Carrette F, Chen J, Lang V, Semichon M, et al (2008) FOXO1 regulates L-Selectin and a network of human T cell homing molecules downstream of phosphatidylinositol 3-kinase. J Immunol 181: 2980–2989. 18713968 10.4049/jimmunol.181.5.2980

[22] Sinclair LV, Finlay D, Feijoo C, Cornish GH, Gray A, et al (2008) Phosphatidylinositol-3-OH kinase and nutrient-sensing mTOR pathways control T lymphocyte trafficking. Nat Immunol 9: 513–521. 18391955 10.1038/ni.1603 PMC2857321

[23] Gerner MY, Kastenmuller W, Ifrim I, Kabat J, Germain RN (2012) Histo-Cytometry: A Method for Highly Multiplex Quantitative Tissue Imaging Analysis Applied to Dendritic Cell Subset Microanatomy in Lymph Nodes. Immunity 37: 1–13. 22863836 10.1016/j.immuni.2012.07.011 PMC3514885

[24] Ohnmacht C, Pullner A, King SB, Drexler I, Meier S, et al (2009) Constitutive ablation of dendritic cells breaks self-tolerance of CD4 T cells and results in spontaneous fatal autoimmunity. J Exp Med 206: 549–559. 19237601 10.1084/jem.20082394 PMC2699126

[25] Steinman RM, Nussenzweig MC (2002) Inaugural Article: Avoiding horror autotoxicus: The importance of dendritic cells in peripheral T cell tolerance. Proc Natl Acad Sci U S A 99: 351–358. 11773639 10.1073/pnas.231606698 PMC117564

[26] Dudziak D, Kamphorst AO, Heidkamp GF, Buchholz VR, Trumpfheller C, et al (2007) Differential antigen processing by dendritic cell subsets in vivo. Science 315: 107–111. 17204652 10.1126/science.1136080

[27] Gunzer M, Schäfer A, Borgmann S, Grabbe S, Zänker KS, et al (2000) Antigen presentation in extracellular matrix: interactions of T cells with dendritic cells are dynamic, short lived, and sequential. Immunity 13: 323–332. 11021530 10.1016/s1074-7613(00)00032-7

[28] Riha P, Rudd CE (2010) CD28 co-signaling in the adaptive immune response. Self Nonself 1: 231–240. 21487479 10.4161/self.1.3.12968 PMC3047785

[29] Crellin NK, Garcia RV, Levings MK (2007) Altered activation of AKT is required for the suppressive function of human CD4+CD25+ T regulatory cells. Blood 109: 2014–2022. 17062729 10.1182/blood-2006-07-035279

[30] Josefowicz SZ, Lu LF, Rudensky AY (2012) Regulatory T cells: mechanisms of differentiation and function. Annu Rev Immunol 30: 531–564. 22224781 10.1146/annurev.immunol.25.022106.141623 PMC6066374

[31] Bilate AM, Lafaille JJ (2012) Induced CD4+Foxp3+ regulatory T cells in immune tolerance. Annu Rev Immunol 30: 733–758. 22224762 10.1146/annurev-immunol-020711-075043

[32] Littman DR, Rudensky AY (2010) Th17 and regulatory T cells in mediating and restraining inflammation. Cell 140: 845–858. 20303875 10.1016/j.cell.2010.02.021

[33] Soond DR, Slack EC, Garden OA, Patton DT, Okkenhaug K (2012) Does the PI3K pathway promote or antagonize regulatory T cell development and function? Front Immunol 3: 244. 22912633 10.3389/fimmu.2012.00244 PMC3418637

[34] Powell JD, Pollizzi KN, Heikamp EB, Horton MR (2012) Regulation of immune responses by mTOR. Annu Rev Immunol 30: 39–68. 22136167 10.1146/annurev-immunol-020711-075024 PMC3616892

[35] Garcon F, Patton DT, Emery JL, Hirsch E, Rottapel R, et al (2008) CD28 provides T-cell costimulation and enhances PI3K activity at the immune synapse independently of its capacity to interact with the p85/p110 heterodimer. Blood 111: 1464–1471. 18006698 10.1182/blood-2007-08-108050

[36] Pletinckx K, Dohler A, Pavlovic V, Lutz MB (2011) Role of dendritic cell maturity/costimulation for generation, homeostasis, and suppressive activity of regulatory T cells. Front Immunol 2: 39. 22566829 10.3389/fimmu.2011.00039 PMC3342346

[37] Guenin-Mace L, Carrette F, Asperti-Boursin F, Le Bon A, Caleechurn L, et al (2011) Mycolactone impairs T cell homing by suppressing microRNA control of L-selectin expression. Proc Natl Acad Sci U S A 108: 12833–12838. 21768364 10.1073/pnas.1016496108 PMC3150933

[38] Levings MK, Gregori S, Tresoldi E, Cazzaniga S, Bonini C, et al (2005) Differentiation of Tr1 cells by immature dendritic cells requires IL-10 but not CD25+CD4+ Tr cells. Blood 105: 1162–1169. 15479730 10.1182/blood-2004-03-1211

[39] Harada Y, Harada Y, Elly C, Ying G, Paik JH, et al.. (2010) Transcription factors Foxo3a and Foxo1 couple the E3 ligase Cbl-b to the induction of Foxp3 expression in induced regulatory T cells. J Exp Med. 10.1084/jem.20100004 PMC2901074 20439537

[40] Kerdiles YM, Stone EL, Beisner DR, McGargill MA, Ch’en IL, et al (2010) Foxo transcription factors control regulatory T cell development and function. Immunity 33: 890–904. 21167754 10.1016/j.immuni.2010.12.002 PMC3034255

[41] Ouyang W, Beckett O, Ma Q, Paik JH, Depinho RA, et al (2010) Foxo proteins cooperatively control the differentiation of Foxp3+ regulatory T cells. Nat Immunol 11: 618–627. 20467422 10.1038/ni.1884

[42] Hedrick SM, Michelini RH, Doedens AL, Goldrath AW, Stone EL (2012) FOXO transcription factors throughout T cell biology. Nat Rev Immunol 12: 649–661. 22918467 10.1038/nri3278 PMC3875397

[43] Dang X, Raffler NA, Ley K (2009) Transcriptional regulation of mouse L-selectin. Biochim Biophys Acta 1789: 146–152. 19041738 10.1016/j.bbagrm.2008.10.004 PMC2650851

[44] Roberts AD, Ely KH, Woodland DL (2005) Differential contributions of central and effector memory T cells to recall responses. J Exp Med 202: 123–133. 15983064 10.1084/jem.20050137 PMC2212898

[45] Yang S, Liu F, Wang QJ, Rosenberg SA, Morgan RA (2011) The shedding of CD62L (L-selectin) regulates the acquisition of lytic activity in human tumor reactive T lymphocytes. PLoS ONE 6: e22560. 21829468 10.1371/journal.pone.0022560 PMC3145643

[46] Battaglia M, Stabilini A, Tresoldi E (2012) Expanding human T regulatory cells with the mTOR-inhibitor rapamycin. Methods Mol Biol 821: 279–293. 22125072 10.1007/978-1-61779-430-8_17

[47] Chen JF, Gao J, Zhang D, Wang ZH, Zhu JY (2010) CD4+Foxp3+ regulatory T cells converted by rapamycin from peripheral CD4+CD25(−) naive T cells display more potent regulatory ability in vitro. Chin Med J (Engl ) 123: 942–948. 20497692

[48] Sauer S, Bruno L, Hertweck A, Finlay D, Leleu M, et al (2008) T cell receptor signaling controls Foxp3 expression via PI3K, Akt, and mTOR. Proc Natl Acad Sci U S A 105: 7797–7802. 18509048 10.1073/pnas.0800928105 PMC2409380

[49] Murphy KM, Heimberger AB, Loh DY (1990) Induction by antigen of intrathymic apoptosis of CD4^+^CD8^+^TCR^lo^ thymocytes in vivo. Science 250: 1720–1723. 2125367 10.1126/science.2125367

[50] Barnden MJ, Allison J, Heath WR, Carbone FR (1998) Defective TCR expression in transgenic mice constructed using cDNA-based alpha- and beta-chain genes under the control of heterologous regulatory elements. Immunol Cell Biol 76: 34–40. 9553774 10.1046/j.1440-1711.1998.00709.x

[51] Labeur MS, Roters B, Pers B, Mehling A, Luger TA, et al (1999) Generation of tumor immunity by bone marrow-derived dendritic cells correlates with dendritic cell maturation stage. J Immunol 162: 168–175. 9886383

[52] Procaccini C, De Rosa V, Galgani M, Abanni L, Cali G, et al (2010) An Oscillatory Switch in mTOR Kinase Activity Sets Regulatory T Cell Responsiveness. Immunity 33: 929–941. 21145759 10.1016/j.immuni.2010.11.024 PMC3133602

