# Supplementary material for: Transiently Reduced PI3K/Akt Activity Drives the Development of Regulatory Function in Antigen-Stimulated Naïve T-Cells
Source: PLoS One. 2013 Jul 11;8(7):e68378. doi: 10.1371/journal.pone.0068378 (PMC3708928; doi:10.1371/journal.pone.0068378)

Figure S1

Effect of inhibitor Titration on CD62-L levels

A

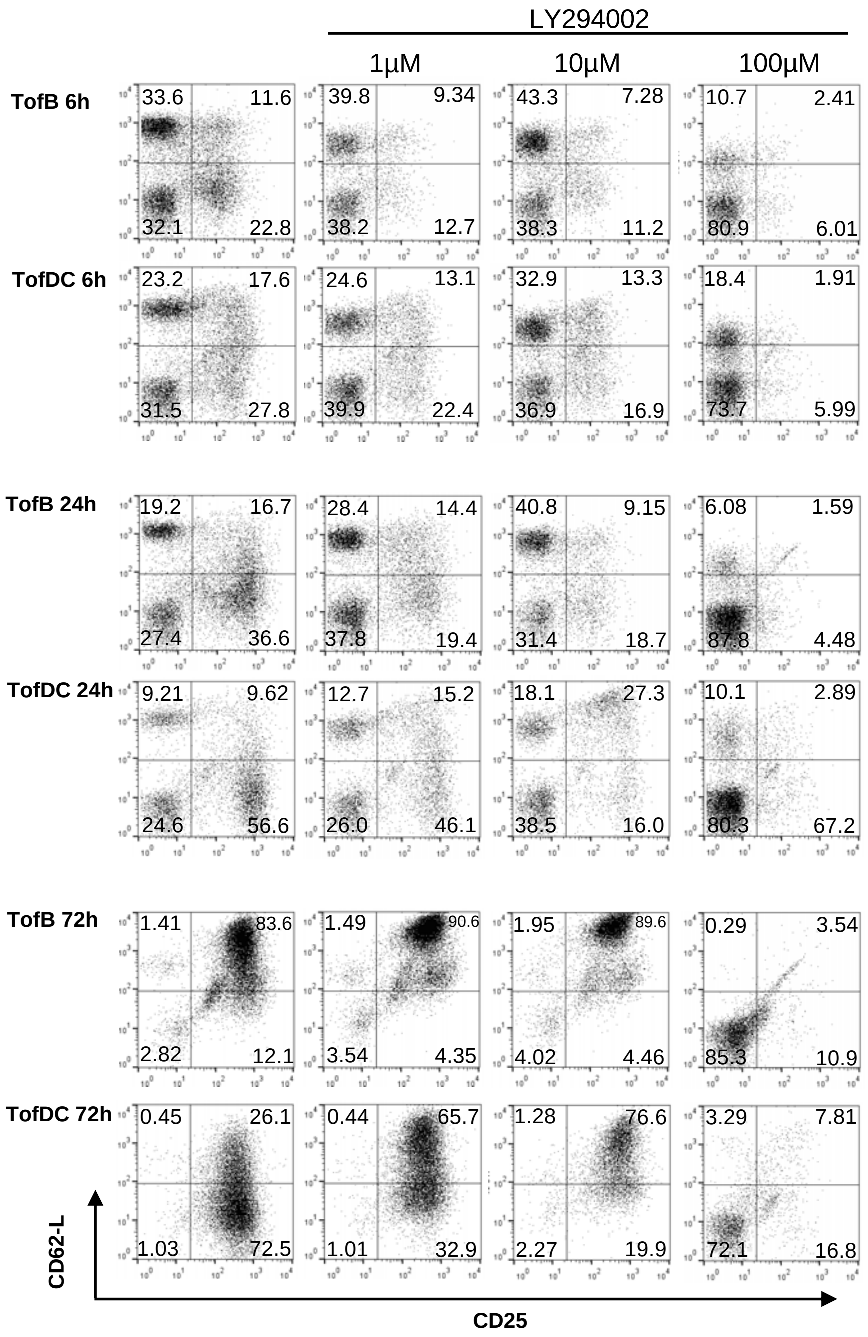

Figure S1 continued

Effect of inhibitor Titration on CD62-L levels

B

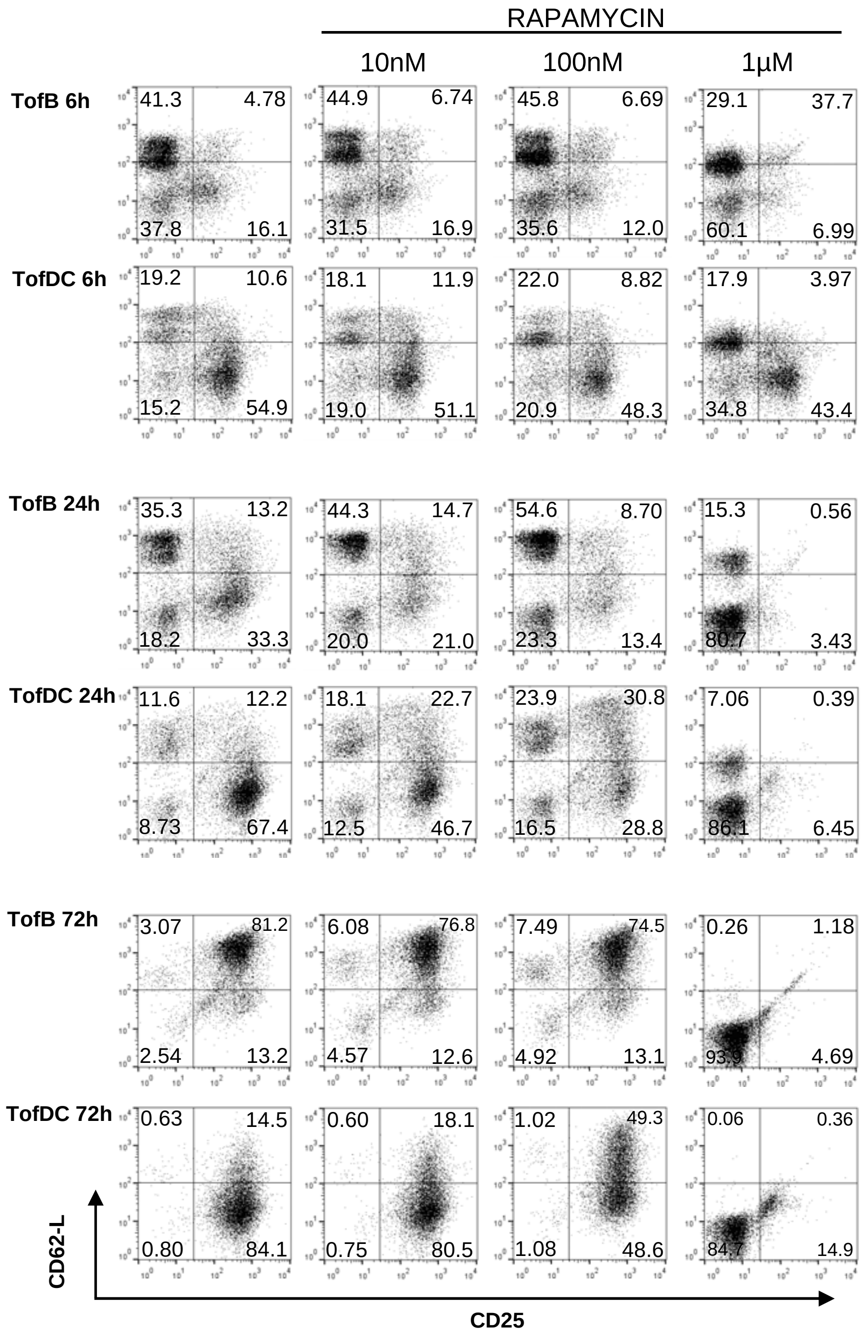

Supplement: Figure S1 — Effect of inhibitor Titration on CD62-L levels. Naïve antigen specific T-cells were stimulated with either naïve B-cells (TofB) or activated dendritic cells (TofDC), both loaded with a cognate peptide of chicken ovalbumin, for different periods of time and in the absence or presence of inhibitors. Subsequently, CD62-L expression levels on T cells were measured by flow cytometry. (A) One representative FACS blot showing the dose dependent regulation of CD62-L in T cells by the PI3K inhibitor LY294002. (B) One representative FACS blot showing the dose dependent regulation of CD62-L in T cells by the mTOR inhibitor Rapamycin. Data are representative of 3 independent experiments. (PDF) [file pone.0068378.s001.pdf]
