# Supplementary figures and images for: Transiently Reduced PI3K/Akt Activity Drives the Development of Regulatory Function in Antigen-Stimulated Naïve T-Cells
Source: PLoS One. 2013 Jul 11;8(7):e68378. doi: 10.1371/journal.pone.0068378 (PMC3708928; doi:10.1371/journal.pone.0068378)

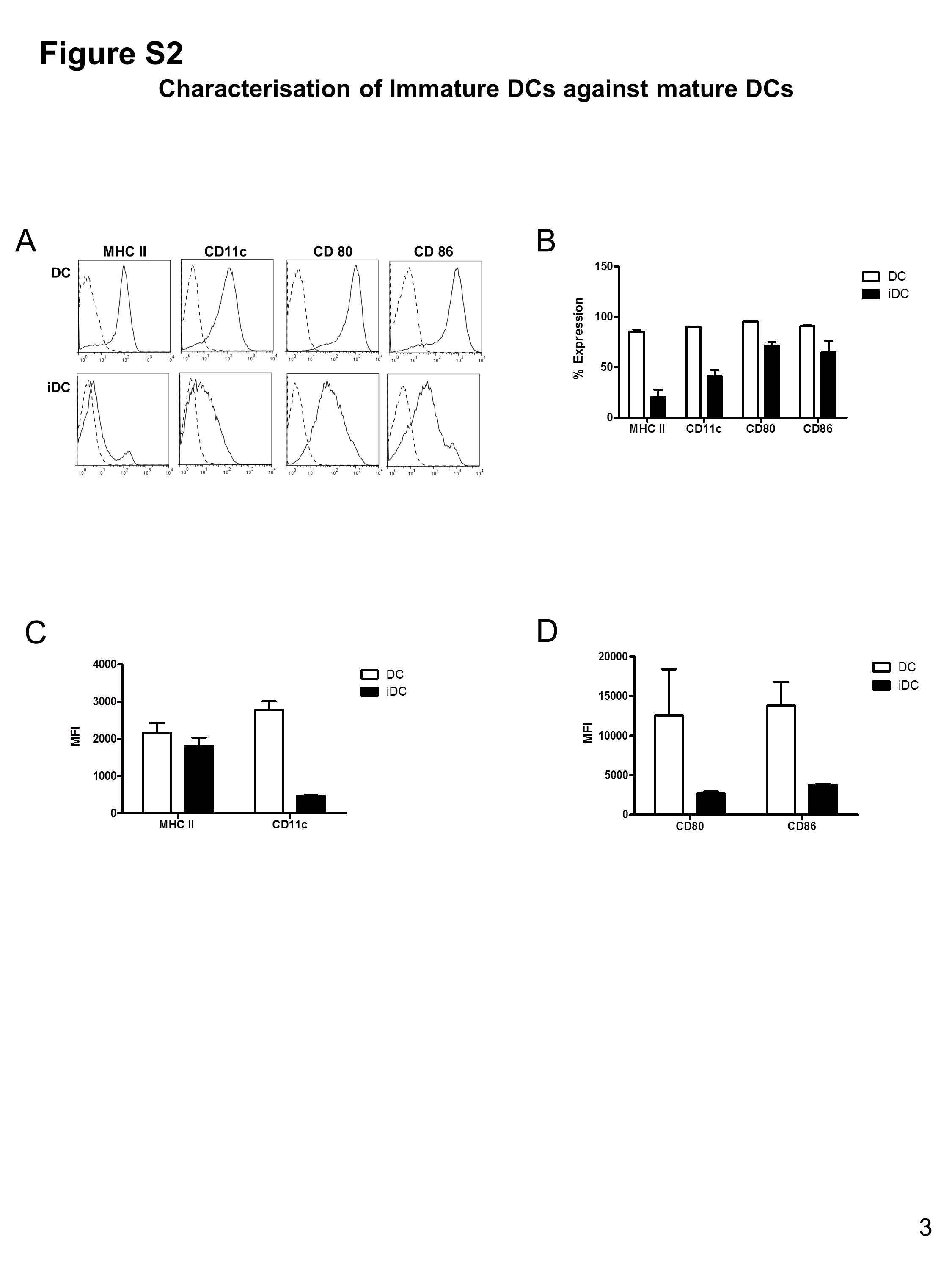

Supplement: Figure S2 — Characterization of Immature DCs against mature DCs. Immature DC were characterized with respect to expression levels of relevant lineage and stimulatory marker molecules.(A) Representative FACS histogram showing expression levels of the indicated surface molecules. Dashed lines represent isotypes and solid lines indicate expression level of quantified molecules (B) Comparison of % surface expression of MHC II, CD11c, CD80 and CD86 between both cell phenotypes. (C) On a per cell basis, DC express more CD11c than immature DC while both cells possess equal amounts of MHC II. (D) On a per cell basis, DC express more co-stimulatory molecules than immature DC. Data are means+SEM of 2 experiments. (TIF) [file pone.0068378.s002.tif]
